# Tolerability of the BNT162b2 COVID-19 Vaccine during Pregnancy among Polish Healthcare Professionals

**DOI:** 10.3390/vaccines10020200

**Published:** 2022-01-27

**Authors:** Wojciech Zdanowski, Agnieszka Markiewicz, Natalia Zdanowska, Janina Lipińska, Tomasz Waśniewski

**Affiliations:** 1 Department of Gynaecology and Obstetrics, Gynaecological Oncology Clinical Ward, Regional Specialist Hospital, ul. Żołnierska 18, 10-561 Olsztyn, Poland; janina.lipinska@uwm.edu.pl (J.L.); tomasz.wasniewski@uwm.edu.pl (T.W.); 2 Department of Obstetrics and Gynaecology, School of Medicine, Collegium Medicum, University of Warmia and Mazury, 10-561 Olsztyn, Poland; 3 Department of Dermatology, Sexually Transmitted Diseases and Clinical Immunology, School of Medicine, Collegium Medicum, University of Warmia and Mazury, 10-229 Olsztyn, Poland; agaen@wp.pl (A.M.); natalia.zdanowska@uwm.edu.pl (N.Z.)

**Keywords:** COVID-19, vaccine, pregnancy

## Abstract

The tolerance and safety of vaccination in pregnancy should be assessed in local populations based on ethnic differences across countries. Therefore, this study aimed to determine the tolerability of the BNT162b2 mRNA vaccination in pregnancy in a Polish population. An online questionnaire enquiring about the safety and tolerability of the BNT162b2 mRNA vaccine was distributed to pregnant and non-pregnant female healthcare professionals who had voluntarily received one or two doses of the COVID-19 vaccine in Poland. The two groups were compared simultaneously considering the COVID-19 infection status before vaccination. Compared with that noted in the control group, pregnant women in the COVID-19-free group were less likely to have fever (*p* = 0.002) or gastrointestinal symptoms (*p* = 0.009) after the second dose. In the COVID-19-exposed group, pregnant women were less likely to experience local skin reactions (*p* = 0.009), and myalgia (*p* = 0.003) after the first dose. After the second dose, the only noticeable difference was a lower incidence of myalgia (*p* = 0.001) in pregnant women. The tolerability of the BNT162b2 mRNA COVID-19 vaccine was similar in both the groups. No severe local, generalised, or pregnancy complications related to mother or foetus were observed. Good tolerability of the BNT162b2 mRNA COVID-19 vaccine in pregnancy in the Polish population may facilitate the decision to vaccinate pregnant women against COVID-19.

## 1. Introduction

Evaluations of the tolerability and safety of coronavirus disease 2019 (COVID-19) vaccination during pregnancy are crucial during the ongoing pandemic. COVID-19 infection is significantly more likely to cause severe disease in pregnant women than in nonpregnant women, with pregnant women being more likely to need oxygen support, respirators, or extracorporeal membrane oxygenation [1,2,3,4,5,6]. 

A growing number of publications demonstrate the safety of COIVD-19 vaccination during pregnancy [7,8,9], but it remains important for researchers to evaluate the safety and tolerability of COVID-19 vaccination during pregnancy in local populations of women. The American College of Obstetricians and Gynecologists, the US Centers for Disease Control and Prevention, the UK Royal College of Obstetricians and Gynaecologists, and the Polish Society of Obstetrics and Gynecology recommend vaccination of pregnant women against COVID-19 [10,11,12,13,14].

In the present study, we explored the tolerability of COVID-19 vaccination in pregnant Polish healthcare professionals. To that end, we conducted an online survey that yielded data from 180 female Polish healthcare professionals who received the BNT162b2 vaccine against COVID-19 while pregnant. We compared their survey responses with those of 40 age-matched female Polish healthcare professionals who received the same vaccine but were not pregnant. 

## 2. Materials and Methods

### 2.1. Study Design and Setting

We developed an online questionnaire containing questions about the tolerability and safety of the BNT162b2 mRNA COVID-19 vaccine. We then distributed the survey by e-mail to pregnant healthcare professionals in Poland who voluntarily received one or two doses of a COVID-19 vaccine between 1 May 2021, and 15 September 2021. The minimum interval between doses was 21 days. We focused on healthcare professionals because healthcare professionals and the elderly were the first groups to receive access to the vaccine in Poland. All survey respondents were ethnically Polish.

All survey respondents gave their informed consent for inclusion before participating in the study. The study was conducted in accordance with the Declaration of Helsinki, and the protocol was approved by the Ethics Com Bioethics Committee of the Medical College of the University of Warmia and Mazury (approval No. 07/2021). 

At the time of data extraction for this study, we had collected survey responses from 284 women who were vaccinated during pregnancy. In addition to the questionnaire responses, we analysed data from the medical records such as age, height, weight, co-morbidities, medications taken, course of pregnancy before and after vaccination.

We excluded 72 women who received the Moderna vaccine, 16 who received the AstraZeneca vaccine, and 16 who received their first dose before pregnancy. These exclusions left us with 180 women who had received one or two doses of the BNT162b2 mRNA COVID-19 vaccine while pregnant. The control group comprised 40 female healthcare professionals who were also vaccinated with the BNT162b2 mRNA COVID-19 vaccine, but were not pregnant in an age range similar to that of the pregnant participants of the study. 

### 2.2. Data Collection and Analysis

The data collected through the survey included basic demographic data, data concerning histories of COVID-19 infection before vaccination (confirmed with polymerase chain reaction [PCR] test results or the presence of antibodies against severe acute respiratory syndrome coronavirus 2 [SARS-CoV-2]), and episodes of quarantine due to contact with a person infected with SARS-CoV-2. These data were confirmed by analysing laboratory test results and medical records. 

The survey respondents were divided into groups based on their histories of exposure to COVID-19. The first of these groups (the COVID-19-free group) included women who had no history of laboratory-confirmed COVID-19 infection before vaccination and no confirmed contact with a COVID-19 infected person. The second group (the COVID-19-exposed group) included women with PCR- or antibody-confirmed histories of COVID-19 infection. The women not assigned to either the COVID-19-free group or the COVID-19-exposed group were those who were quarantined after contact with an infected person but had not tested positive for COVID-19.

A local reaction was defined as pain or local erythema at the site of vaccination. Generalised symptoms included fever, headache, gastrointestinal symptoms, myalgia, dyspnoea, and fatigue. Each symptom was also evaluated in terms of the time of onset after vaccination with the first dose or second dose.

### 2.3. Statistical Analysis

Statistical analyses were performed using Statistica software (v13.3, StatSoft, Kraków, Poland). Data expressed on a qualitative scale were presented as the number and percentage of the sample. The Chi-squared test (χ2) was used to compare the relationships between variables expressed in the qualitative scale. Data expressed on a quantitative scale were presented as the mean with standard deviation (SD) values. As the data were not normally distributed (Shapiro–Wilk test), the Mann–Whitney test were used. Results were considered statistically significant if *p* < 0.05.

## 3. Results

### 3.1. Characteristics of the Survey Respondents

The COVID-19-free group included 145 pregnant women and 19 non-pregnant women (control group). There was no difference between pregnant and control subgroups in terms of age (*p* = 0.242) and body mass index (BMI) values (*p* = 0.098) among those who were COVID-19-free. Among them, 105 (72.4%) pregnant and 18 (94.7%) non-pregnant women had received both doses of vaccine.

The COVID-19-exposed group included 24 pregnant (pregnancy group) and 16 non-pregnant women (control group). In the COVID-19-exposed group, women in the pregnancy and control subgroups were similar in terms of age (*p =* 0.242) and BMI (*p =* 0.098). Sixteen (66.7%) women in the pregnancy subgroup, and 15 (93.8%) women in the control subgroup had received both doses. One woman in the pregnancy subgroup was carrying twins, while the others were carrying singleton pregnancies. 

### 3.2. Tolerability of the First Dose of the BNT162b2 mRNA COVID-19 Vaccine in the COVID-19-Free Group

Table 1 summarizes the adverse events observed in the COVID-19-free group after the first vaccine dose. In the COVID-19-free group, 138 (94.5%) pregnant women and 19 (100%) control women experienced local or generalised pain after receiving the first dose (*p* = 0.271). The pregnancy and the control subgroups were similar in terms of the percentages of women experiencing vaccine site pain (*p =* 0.271) and local cutaneous reactions at the vaccine site (*p* = 0.464) after the first dose. They were also similar in terms of the percentages of women experiencing headache (*p =* 0.608), myalgia (*p =* 0.420), fever (*p =* 0.234), fatigue (*p =* 0.701), and gastrointestinal symptoms (*p =* 0.061) in the week after the first dose. Nausea was the only gastrointestinal symptom reported.

### 3.3. Tolerability of the Second Dose of the BNT162b2 mRNA COVID-19 Vaccine in the COVID-19-Free Group

Table 1 summarizes the adverse events observed in the COVID-19-free group after the second vaccine dose. In the COVID-19-free group, 93 (87.7%) pregnant women and 18 (100%) control women experienced local or generalised symptoms after receiving the second dose (*p* = 0.106). The pregnancy and the control subgroups were similar in terms of the percentages of women experiencing vaccine site pain (*p =* 0.106) and local cutaneous reactions at the vaccine site (*p =* 0.729). They were also similar in terms of the percentages of women experiencing headache (*p =* 0.630), myalgia (*p =* 0.427), and fatigue (*p =* 0.521) in the week after the first dose. However, the women in the pregnancy group were less likely to experience fever (*p =* 0.019) and gastrointestinal symptoms (*p =* 0.009) during the first week after the second dose. The reported gastrointestinal symptoms included nausea, vomiting, diarrhoea, and abdominal pain.

### 3.4. Tolerability of the First Dose of the BNT162b2 mRNA COVID-19 Vaccine in the COVID-19-Exposed Group

Table 2 summarizes the adverse events observed in the COVID-19-exposed group after the first vaccine dose. Within the COVID-19-exposed group, 24 (100%) pregnant women and 16 (100%) control women experienced local or generalised symptoms after receiving the first dose (*p* = 1). All women in the COVID-19-exposed group experienced vaccine site pain. Compared to the control women, pregnant women were less likely to experience cutaneous reactions at the vaccine site (*p* = 0.009). The pregnancy and control subgroups were similar in terms of the percentages of women who experienced headache (*p* = 0.134), fatigue (*p* = 0.727), and gastrointestinal symptoms (*p* = 0.206) in the week after the first dose. Compared to women in the control women, pregnant women were less likely to experience fever (*p* = 0.013) and myalgia (*p* = 0.028) in the week after the first dose.

### 3.5. Tolerability of the Second Dose of the BNT162b2 mRNA COVID-19 Vaccine in the COVID-19-Exposed Group

Table 2 summarizes the adverse events observed in the COVID-19-exposed group after the second vaccine dose. In the COVID-19-exposed group, 14 (87.5%) pregnant women and 13 (86.7%) control women experienced local or generalised after receiving the second dose (*p* = 0.944). The pregnancy and control subgroups were similar in terms of the percentages of women experiencing vaccine site pain lasting (*p* = 0.944) and cutaneous reactions at the vaccine site (*p* = 0.319). The pregnancy and control subgroups were similar in terms of the percentages of women experiencing headache (*p* = 0.160), fever (*p* = 0.170), fatigue (*p* = 0.734), and gastrointestinal symptoms (*p* = 0.684) in the week after the second dose. Compared to the control women, pregnant women were less likely to experience myalgia (*p* = 0.011) in the week after the second dose. 

## 4. Discussion

Evaluations of the tolerability and safety of vaccination during pregnancy provide key information for officials who decide whether pregnant woman should receive vaccinations against COVID-19. This question is an important one, especially in light of the current evidence that pregnant women are at an increased risk of developing severe COVID-19 [1,2,3,4,5,6]. For obvious reasons, pregnant women are not a group in which clinical trials evaluating the safety of vaccination are conducted, and they were not included in trials of COVID-19 vaccines until phase III trials were completed [15]. It is therefore important to collect and analyse data from women who voluntarily received vaccinations during pregnancy. The evidence from the present study suggests that the BNT162b2 mRNA COVID-19 vaccine is safe and tolerable for women during pregnancy.

There were no severe local or generalised complications or severe maternal or foetus related pregnancy complications in our study’s pregnancy group. An increasing number of studies have confirmed the safety of COVID-19 vaccination in pregnant women [16,17,18], and other studies have also confirmed the good tolerability of the BNT162b2 mRNA COVID-19 vaccine in the general population [19,20]. Our findings showed that the safety and tolerability of COVID-19 vaccination during pregnancy holds true for the Polish population.

Side effects are natural responses to the injection of a foreign object and can involve symptoms such as fever, muscle aches, and inflammation at the injection site. These symptoms are triggered by the innate immune system. When the body’s neutrophils or macrophages detect vaccine molecules, they release cytokines, which are chemical messengers that induce immune responses in the form of fever, nausea, and muscle aches. Such cytokine responses can be observed when a foreign agent is injected into the circulatory system [21].

The survey respondents in this study were divided into two groups. The first group consisted of women without histories of PCR- or antibody-confirmed COVID-19 infection and without any known contact with a COVID-19–infected person. The second group consisted of women with histories of PCR- or antibody-confirmed COVID-19 infection. Knowing a survey respondent’s history of COVID-19 infection may be important in the evaluation of vaccine tolerability during pregnancy because studies published to date have shown poorer vaccine tolerability in individuals with a history of COVID-19 infection, which mainly manifests in response to the first dose of the BNT162b2 mRNA COVID-19 vaccine [22,23,24].

In our COVID-19-free group, there was no statistically significant difference in the reported adverse events after the first dose of the BNT162b2 mRNA COVID-19 vaccine between pregnant and control women. In fact, pregnant COVID-19-free women were less likely to develop fever or gastrointestinal symptoms after the second dose of the vaccine. During pregnancy, many complex processes leading to immunotolerance occur, including a shift in the T lymphocyte response toward Th1 cells, the generation of a temporal subpopulation of natural killer cells, and the placental production of many immunomodulatory substances such as neurokinin B, interleukin-10, and interleukin-35 [25,26,27]. The purpose of these changes is to ensure the normal development of pregnancy by inhibiting the graft-versus-host response while providing an anti-infective defence in pregnant woman [26]. It is possible that these alterations influenced the aforementioned diminished frequency of certain vaccine side effects during pregnancy. There are currently no other publications available that similarly compare pregnant and nonpregnant women in terms of side effects after the administration of mRNA vaccines. 

Within our COVID-19–exposed group, pregnant women were less likely to experience symptoms such as local skin reactions, and myalgia after the first dose than control women were. After the second dose, the only observable difference was a lower frequency of myalgia in pregnant women. No significant differences were observed in other symptoms. 

It is worth noting that in the COVID-19-exposed group, the tolerability of the first dose was better than in the COVID-19-exposed non-pregnant group. No such difference in first-dose tolerability was shown in the COVID-19-free pregnant group compared with controls. As the first dose is less well tolerated after COVID-19 infection in the general population it is likely that the mechanisms of immunotolerance are probably more responsible in reducing the number of adverse events among COVID-19-exposed pregnant women than in the COVID-19-free group [22,23,24,28]. There is a lack of information in the current literature regarding the effect of COVID-19 infection on the immune system in pregnant women. 

The pregnancy and control groups did not significantly differ in terms of the occurrence of fatigue. None of the patients experienced bleeding or spotting per vaginum for up to seven days after vaccination. One woman reported short-term dyspnoea after the second dose, but she had a previous diagnosis of polyhydramnios, no one develops general allergic reaction. 

In the present study, we found no significant difference in the total number of adverse reactions between pregnant and control groups. The tolerability of the second dose was worse for both the pregnancy and control groups, with the rate of adverse events increasing by 13.6% in the pregnancy group and by 10% in the control group. This finding is consistent with the findings of previous studies comparing the frequency of side effects after the first and second doses of the BNT162b2 mRNA COVID-19 vaccine [29,30]. However, these studies did not include pregnant women.

In the present study, we found that tolerability of the BNT162b2 mRNA COVID-19 vaccine is similar in the study and control groups and that some adverse reactions were less frequent in pregnant women than in nonpregnant women. These findings may help in encouraging women to receive COVID-19 vaccination during pregnancy. Vaccination not only reduces the risk of severe COVID-19 in pregnant women, but also reduces the risk of infection in their infants through the placental transfer of antibodies [31,32,33].

The present study’s limitations include its small sample size, small control group and its retrospective nature. Thorough evaluations of vaccine tolerability will require further follow-up with a larger population of pregnant and non-pregnant women in Poland.

## 5. Conclusions

The tolerability and safety of COVID-19 vaccination for pregnant women are key issues due to the ongoing pandemic, especially given the fact that pregnant women are more severely affected by COVID-19. In our study, we demonstrated the tolerability of the BNT162b2 mRNA COVID-19 vaccine for pregnant women in the Polish population. Good tolerability of the BNT162b2 mRNA COVID-19 vaccine in pregnancy in the Polish population may facilitate the decision to vaccinate pregnant women against COVID-19. No severe local or generalised complications among either the mothers or the foetuses were observed. 

## Figures and Tables

**Table 1:** Tolerability of the first and the second doses of the BNT162b2 mRNA COVID-19 vaccine in the COVID-19–free group.

	Pregnant Women COVID-19 −	Non-Pregnant Women COVID-19 −	*p*	Total COVID-19 −
Age, mean (SD)	31.4 (+/−2.6) y	33.1 (+/−3.6) y	0.242 ^#^	N/A
BMI, mean (SD)	22.75 (+/−3.59) kg/m^2^	21.7 (+/−3.38) kg/m^2^	0.098 ^#^	N/A
One dose of the vaccine administered, *n* (%)	145 (100%)	19 (100%)	N/A	164 (100%)
Two doses of the vaccine administered, *n* (%)	105 (72.4%)	18 (94.7%)	N/A	123 (75%)
Side effects after first dose				
Vaccination site pain total, *n* (%)	138 (94.5%)	19 (100%)	0.271 ^^^	157 (95.7%)
Cutaneous reaction total, *n* (%)	14 (9.7%)	1 (4.8%)	0.464 ^^^	15 (9.1%)
Headache total, *n* (%)	16 (11.0%)	3 (14.3%)	0.608 ^^^	19 (11.6%)
On the first day, *n* (%)	13 (9.0%)	3 (14.3%)		16 (9.8%)
Between the second and seventh days, *n* (%)	3 (2.1%)	None		3 (1.8%)
Fever			0.234 ^^^	
On the first day, *n* (%)	2 (1.4%)	1 (5.3%)		3 (1.8%)
Between the second and seventh days, *n* (%)	None	None		None
Myalgia			0.420 ^^^	
On the first day, *n* (%)	2 (1.4%)	1 (4.8%)		3 (1.8%)
Between the second and seventh days, *n* (%)	3 (2.1%)	1 (4.8%)		4 (2.4%)
Fatigue up to the seventh day, *n* (%)	51 (35.2%)	6 (31.5%)	0.701 ^^^	57 (34.7%)
Gastrointestinal symptoms up to the seventh day			0.061 ^^^	
Total, *n* (%)	3 (2.1%)	2 (9.5%)		5 (3.0%)
Nausea, *n* (%)	3 (2.1%)	2 (9.5%)		5 (3.0%)
Vomiting, *n* (%)	None	None		None
Diarrhoea, *n* (%)	None	None		None
Side effects after second dose				
Vaccination site pain total, *n* (%)	93 (87.7%)	18 (100)	0.106 ^^^	21 (89.4%)
Cutaneous reaction total, *n* (%)	7 (6.7%)	1 (5.3%)	0.729 ^^^	8 (6.5%)
Headache total, *n* (%)	42 (31.4%)	8 (47.4%)	0.630 ^^^	50 (40.6%)
On the first day, *n* (%)	33 (31.4%)	8 (42.1%)		41 (33.3%)
Between the second and seventh days, *n* (%)	9 (8.6%)	1 (5.3%)		10 (8.1%)
Fever			0.019 *^^^	
On the first day, *n* (%)	17 (16.2%)	8 (42.1%)		25 (20.3%)
Between the second and seventh days, *n* (%)	2 (1.9%)	1 (5.3%)		3 (2.4%)
Myalgia			0.427 ^^^	
On the first day, *n* (%)	29 (27.6%)	8 (41.1%)		37 (30.1%)
Between the second and seventh days, n (%)	11 (10.5%)	2 (10.5%)		13 (10.6%)
Fatigue up to the seventh day, *n* (%)	54 (51.4%)	8 (41.1%)	0.521 ^^^	62 (50.4%)
Gastrointestinal symptoms up to the seventh day			0.009 *^^^	
Total, *n* (%)	9 (8.6%)	6 (31.6%)		15 (12.2%)
Nausea, *n* (%)	4 (3.8%)	3 (15.8%)		7 (5.7%)
Vomiting, *n* (%)	3 (2.9%)	None		3 (2.4%)
Diarrhoea, *n* (%)	2 (1.9%)	3 (15.8%)		5 (4.1%)

* *p* < 0.05; BMI, body mass index; SD, standard deviation; ^#^ Mann–Whitney test; ^^^ Chi-squared test.

**Table 2:** Tolerability of the first and the second doses of the BNT162b2 mRNA COVID-19 vaccine in the COVID-19–exposed group.

	Pregnant Women COVID-19 +	Non-Pregnant Women COVID-19 +	*p*	Total COVID-19 +
Age, mean (SD)	31.25 (+/−1.9)) y	34 (+/−4.4) y	0.242 ^#^	N/A
BMI, mean (SD)	21.7 (+/−3.75) kg/m^2^	22.9 (+/−3.73) kg/m^2^	0.098 ^#^	N/A
One dose of the vaccine administered, *n* (%)	24 (100%)	16 (100%)	N/A	40 (100%)
Two doses of the vaccine administered, *n* (%)	16 (66.7%)	15 (93.6%)	N/A	31 (77.5%)
Side effects after first dose				
Vaccination site pain total, *n* (%)	24 (100%)	16 (100%)	1 ^^^	40 (100%)
Cutaneous reaction total, *n* (%)	None	4 (25.0%)	0.009 *^^^	4 (10.0%)
Headache total, *n* (%)	1 (4.2%)	4 (25.0%)	0.134 ^^^	5 (12.5%)
On the first day, *n* (%)	1 (4.2%)	3 (18.8%)		
Between the second and seventh days, *n* (%)	None	1 (6.3%)		
Fever			0.013 ^^^	
On the first day, *n* (%)	None	2 (12.5%)		2 (5%)
Between the second and seventh days, *n* (%)	None	3 (18.8%)		3 (7.5%)
Myalgia			0.028 *^^^	
On the first day, *n* (%)	1 (4.2%)	5 (31.3%)		6 (15.0%)
Between the second and seventh days, *n* (%)	1 (4.2%)	2 (12.5%)		3 (7.5%)
Fatigue up to the seventh day, *n* (%)	10 (41.7%)	6 (37.5%)	0.727 ^^^	16 (40.0%)
Gastrointestinal symptoms up to the seventh day			0.206 ^^^	
Total, *n* (%)	None	2 (12.5%)		2 (5%)
Nausea, *n* (%)	None	1 (6.3%)		1 (2.5%)
Vomiting, *n* (%)	None	1 (6.3%)		1 (2.5%)
Diarrhoea, *n* (%)	None	None		None
Side effects after second dose				
Vaccination site pain, *n* (%)	14 (87.5%)	13 (86.7%)	0.944 ^^^	27 (87.0%)
Cutaneous reaction total, *n* (%)	None	2 (13.3%)	0.319 ^^^	2 (6.5%)
Headache total, *n* (%)	2 (12.5%)	4 (26.7%)	0.160 ^^^	6 (19.4%)
On the first day, *n* (%)	2 (12.5%)	1 (6.7%)		
Between the second and seventh days, *n* (%)	None	3 (20.0%)		
Fever			0.170 ^^^	
On the first day, *n* (%)	None	1 (6.7%)		1 (3.2%)
Between the second and seventh days, *n* (%)	None	2 (13.3%)		2 (6.5%)
Myalgia			0.011 *^^^	
On the first day, *n* (%)	1 (6.3%)	3 (20.0%)		4 (12,9)
Between the second and seventh days, *n* (%)	None	5 (33.0%)		5 (16,13)
Fatigue up to the seventh day, *n* (%)	7 (43.8%)	8 (53.3%)	0.734 ^^^	15 (48)
Gastrointestinal symptoms up to the seventh day			0.684 ^^^	
Total, *n* (%)	None	2 (13.3%)		2 (6.5%)
Nausea, *n* (%)	None	1 (6.7%)		1 (3.2%)
Vomiting, *n* (%)	None	None		None
Diarrhoea, *n* (%)	None	1 (6.7%)		1 (3.2%)

* *p* < 0.05; BMI, body mass index; SD, standard deviation; ^#^ Mann–Whitney test; ^^^ Chi-squared test.

## Data Availability

Paper and digital data are available for review upon request.

## References

[1] Wang C.L., Liu Y.Y., Wu C.H., Wang C.Y., Wang C.H., Long C.Y. (2021). Impact of COVID-19 on Pregnancy. Int. J. Med. Sci..

[2] Salem D., Katranji F., Bakdash T. (2021). COVID-19 infection in pregnant women: Review of maternal and fetal outcomes. Int. J. Gynaecol. Obstet..

[3] Jafari M., Pormohammad A., Sheikh Neshin S.A., Ghorbani S., Bose D., Alimohammadi S., Basirjafari S., Mohammadi M., Rasmussen-Ivey C., Razizadeh M.H. (2021). Clinical characteristics and outcomes of pregnant women with COVID-19 and comparison with control patients: A systematic review and meta-analysis. Rev. Med. Virol..

[4] De Oliveira K.F., de Oliveira J.F., Wernet M., Carvalho Paschoini M., Ruiz M.T. (2021). COVID-19 and pregnancy: A scoping review on pregnancy characteristics and outcomes. Int. J. Nurs. Pract..

[5] Wei S.Q., Bilodeau-Bertrand M., Liu S., Auger N. (2021). The impact of COVID-19 on pregnancy outcomes: A systematic review and meta-analysis. CMAJ.

[6] Kotlyar A.M., Grechukhina O., Chen A., Popkhadze S., Grimshaw A., Tal O., Taylor H.S., Tal R. (2021). Vertical transmission of coronavirus disease 2019: A systematic review and meta-analysis. Am. J. Obstet. Gynecol..

[7] Donders G.G.G., Grinceviciene S., Haldre K., Lonnee-Hoffmann R., Donders F., Tsiakalos A., Adriaanse A., Martinez de Oliveira J., Ault K., Mendling W. (2021). On The behalf of the Covid-Isidog Guideline Group. ISIDOG Consensus Guidelines on COVID-19 vaccination for women before, during and after Pregnancy. J. Clin. Med..

[8] Shook L.L., Fallah P.N., Silberman J.N., Edlow A.G. (2021). COVID-19 Vaccination in Pregnancy and Lactation: Current research and gaps in understanding. Front. Cell Infect. Microbiol..

[9] Luxi N., Giovanazzi A., Capuano A., Crisafulli S., Cutroneo P.M., Fantini M.P., Ferrajolo C., Moretti U., Poluzzi E., Raschi E. (2021). Ilmiovaccino COVID19 collaborating group. COVID-19 vaccination in pregnancy, paediatrics, immunocompromised patients, and persons with history of allergy or prior SARS-CoV-2 infection: Overview of current recommendations and pre- and post-marketing evidence for vaccine efficacy and safety. Drug Saf..

[10] (2021). ACOG Vaccinating Pregnant and Lactating Patients against COVID-19. https://www.acog.org/clinical/clinical-guidance/practice-advisory/articles/2020/12/vaccinating-pregnant-and-lactating-patients-against-covid-19.

[11] COVID-19 (Coronavirus Disease): People with Certain Medical Conditions. https://www.cdc.gov/coronavirus/2019-ncov/need-extra-precautions/people-with-medical-conditions.html.

[12] COVID-19 (Coronavirus Disease): Information about COVID-19 Vaccines for People Who Are Pregnant or Breastfeeding. https://www.cdc.gov/coronavirus/2019-ncov/vaccines/recommendations/pregnancy.html.

[13] Royal College of Obstetricians and Gynaecologists Updated Advice on COVID-19 Vaccination in Pregnancy and Women Who Are Breastfeeding. https://www.rcog.org.uk/en/news/updated-advice-on-covid-19-vaccination-in-pregnancy-and-women-who-are-breastfeeding/.

[14] PTGiP Position Statement on Vaccination of Pregnant Women against COVID19. https://www.ptgin.pl/sites/scm/files/2021-09/04.2021%20Stanowisko%20PTGiP%20dotyczące%20szczepień%20kobiet%20ciężarnych%20przeciwko%20COVID19.pdf.

[15] Damasceno D.H.P., Amaral A.A., Silva C.A., Simões E., Silva A.C. The Impact of Vaccination Worldwide on SARS-CoV-2 Infection: A Review on Vaccine Mechanisms, Results of Clinical Trials, Vaccinal Coverage and Interactions with Novel Variants. https://pubmed.ncbi.nlm.nih.gov/34473613/.

[16] Wainstock T., Yoles I., Sergienko R., Sheiner E. (2021). Prenatal maternal COVID-19 vaccination and pregnancy outcomes. Vaccine.

[17] Burd I., Kino T., Segars J. (2021). The Israeli study of Pfizer BNT162b2 vaccine in pregnancy: Considering maternal and neonatal benefits. J. Clin. Investig..

[18] Morales-Núñez J.J., Muñoz-Valle J.F., Meza-López C., Wang L.F., Machado Sulbarán A.C., Torres-Hernández P.C., Bedolla-Barajas M., De la O-Gómez B., Balcázar-Félix P., Hernández-Bello J. (2021). Neutralizing antibodies titers and side effects in response to BNT162b2 vaccine in healthcare workers with and without prior SARS-CoV-2 infection. Vaccines.

[19] Polack F.P., Thomas S.J., Kitchin N., Absalon J., Gurtman A., Lockhart S., Perez J.L., Pérez Marc G., Moreira E.D., Zerbini C. (2020). C4591001 Clinical Trial Group. Safety and Efficacy of the BNT162b2 mRNA COVID-19 Vaccine. N. Engl. J. Med..

[20] Baden L.R., El Sahly H.M., Essink B., Kotloff K., Frey S., Novak R., Diemert D., Spector S.A., Rouphael N., Creech C.B. (2021). COVE Study Group. Efficacy and safety of the mRNA-1273 SARS-CoV-2 vaccine. N. Engl. J. Med..

[21] Vogel W.H. (2010). Infusion reactions: Diagnosis, assessment, and management. Clin. J. Oncol. Nurs..

[22] Theiler R.N., Wick M., Mehta R., Weaver A.L., Virk A., Swift M. (2021). Pregnancy and birth outcomes after SARS-CoV-2 vaccination in pregnancy. Am. J. Obstet. Gynecol. MFM.

[23] Raw R.K., Rees J., Kelly C.A., Wroe C., Chadwick D.R. (2021). Prior COVID-19 infection is associated with increased adverse events (AEs) after the first, but not the second, dose of the BNT162b2/Pfizer vaccine. Vaccine.

[24] Baldolli A., Michon J., Appia F., Galimard C., Verdon R., Parienti J.J. (2021). Tolerance of BNT162b2 mRNA COVI-19 vaccine in patients with a medical history of COVID-19 disease: A case control study. Vaccine.

[25] Orefice R. (2021). Immunology and the immunological response in pregnancy. Best Pract. Res. Clin. Obstet. Gynaecol..

[26] Cornish E.F., Filipovic I., Åsenius F., Williams D.J., McDonnell T. (2020). Innate immune responses to acute viral infection during pregnancy. Front. Immunol..

[27] Zdanowska N., Owczarczyk-Saczonek A.B., Zdanowski W., Placek W.J. (2020). Interleukin 35: An overview. Pol. Ann. Med..

[28] Krammer F., Srivastava K., Alshammary H., Amoako A.A., Awawda M.H., Beach K.F., Bermúdez-González M.C., Bielak D.A., Carreño J.M., Chernet R.L. (2021). Antibody Responses in Seropositive Persons after a Single Dose of SARS-CoV-2 mRNA Vaccine. N. Engl. J. Med..

[29] Andrzejczak-Grządko S., Czudy Z., Donderska M. (2021). Side effects after COVID-19 vaccinations among residents of Poland. Eur. Rev. Med. Pharmacol. Sci.

[30] Lee Y.W., Lim S.Y., Lee J.H., Lim J.S., Kim M., Kwon S., Joo J., Kwak S.H., Kim E.O., Kwon H.S. (2021). Adverse reactions of the second dose of the BNT162b2 mRNA COVID-19 vaccine in healthcare workers in Korea. J. Korean Med. Sci..

[31] Kim L., Whitaker M., O’Halloran A., Kambhampati A., Chai S.J., Reingold A., Armistead I., Kawasaki B., Meek J., Yousey-Hindes K. (2020). Hospitalization rates and characteristics of children aged <18 Years hospitalized with laboratory-confirmed COVID-19 COVID-NET, 14 States, March 1–July 25, 2020. Morb Mortal Wkly Rep..

[32] Beharier O., Mayo R.P., Raz T., Sacks K.N., Schreiber L., Suissa-Cohen Y., Chen R., Gomez-Tolub R., Hadar E., Gabbay-Benziv R. (2021). Efficient maternal to neonatal transfer of antibodies against SARS-CoV-2 and BNT162b2 mRNA COVID-19 vaccine. J. Clin. Investig..

[33] Zdanowski W., Waśniewski T. (2021). Evaluation of SARS-CoV-2 spike protein antibody titers in cord blood after COVID-19 vaccination during pregnancy in Polish healthcare workers: Preliminary results. Vaccines.

